# A Rare Case of Primary Solid Intrarectal Dermoid

**DOI:** 10.4103/1319-3767.74466

**Published:** 2011

**Authors:** Onkar Singh, Shilpi S. Gupta, Raj K. Mathur

**Affiliations:** Department of Surgery, MGM Medical College and MY Hospital, Indore - 452 001, India

**Keywords:** Dermoid cyst, mature teratoma, rectum

## Abstract

Primary rectal teratomas are rare and only few cases have been reported in the literature worldwide. These usually occur in females. These are usually cystic but very rarely solid variants may occur. We present a case of a solid intra-rectal dermoid arising primarily in rectum from postero-lateral wall. Excision biopsy was done per rectally. Histology revealed the presence of squamous epithelium, fat cells, hair follicles, cartilaginous material and columnar lining of glandular structures suggestive of mature teratoma. It is usually benign but may become malignant, therefore complete resection is advised.

Rectal cysts are uncommon and primary rectal dermoid is exceedingly rare; only 36 cases have been reported in the literature worldwide.[1] Intrarectal dermoid is most commonly found in females. These are believed to develop as a result of continued growth of trapped ectodermal tissue.[1,2] Though it is also presumed that anterior rectal dermoids arise in the ovary and erode into the rectum, up to date there has been no substantial evidence of this.[3] We present a case of a primary intra rectal solid dermoid arising from postero-lateral rectal wall, which is very rare.

## CASE REPORT

A 29-year-old, otherwise healthy female patient presented with complains of progressively increasing pain on defecation with minimal bleeding per rectum for last three months. There was no history of loss of appetite or weight loss. General physical examination was unremarkable. Abdominal examination did not show any abnormality. Per rectal digital examination revealed a 6 × 5 cm sized solid tumor located about 5 cm from anal verge and protruding in to rectal lumen with stalk arising from postero-lateral rectal wall.

Sigmoidoscopy revealed single polypoidal tumor of size 6 × 5 × 4 cm with a stalk of size 3 cm arising from postero-lateral wall of rectum. The surface was smooth and glistening. There was no evidence of any other growth or synchronous polyp on colonoscopy. Computerized tomography scan of the abdomen and pelvis also revealed a well localized intra luminal growth in the rectum arising from the posterolateral wall with no evidence of extension in to adjacent organs. Both the ovaries were normal.

**Figure 1:**
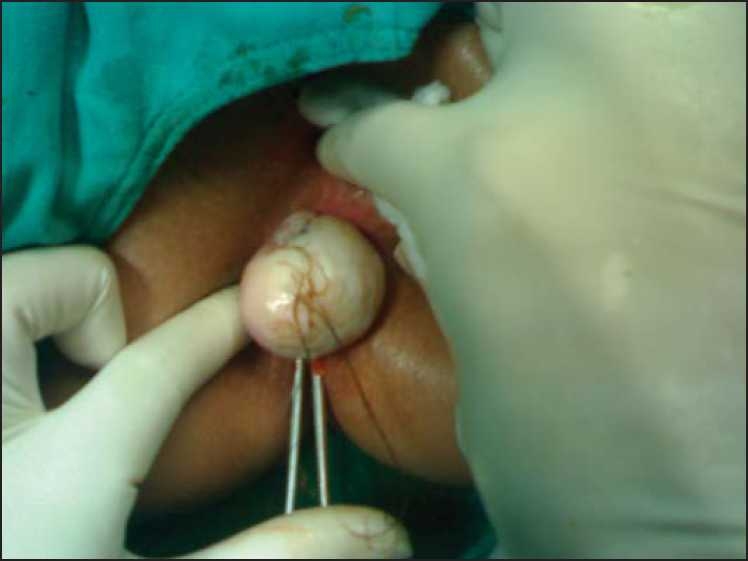
Solid intra-rectal dermoid being delivered out of the rectum

**Figure 2:**
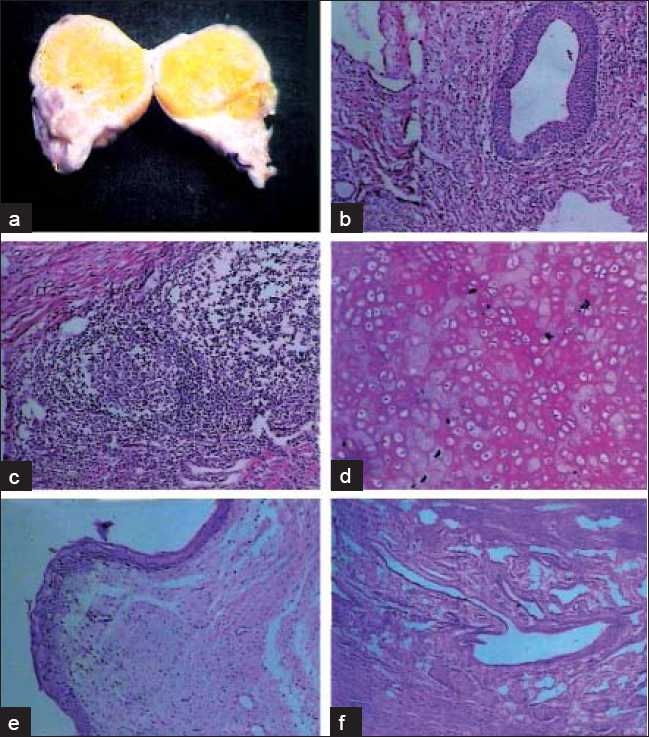
(a-f) Cut section and histopathology showing features of mature solid teratoma

Excision biopsy was planned per rectally. Intra-operatively, the tumor was delivered per-anal [Figure 1]. Stalk of the tumor was transfixed and cut near its base. Grossly, the tumor was solid and 5.5 × 4 × 3 cm in size. It had smooth glistening surface. Cut section showed yellowish firm areas. Histological examination revealed the presence of squamous epithelium, fat cells, hair follicles, cartilaginous material and columnar lining of glandular structures suggestive of mature teratoma. There was no evidence of immature tissue or malignancy at the base of stalk [Figure 2].

## DISCUSSION

Dermoid cysts are a special form of mature teratoma that predominately have an ectodermal derivation; although, structures derived from ectoderm, mesoderm and endoderm are all commonly represented.[4] These have been commonly reported in the ovaries, testes, and mediastinum, and are less commonly reported to involve various midline locations, including the sacrococcygeal area. These are rarely found in the neuraxis, spermatic cord, or gastrointestinal tract, including the floor of the mouth, rectum, sigmoid colon, appendix, and terminal ileum. The gastrointestinal tract is an unusual site for teratomas to occur, although it has been described to affect caecum.[4] Primary rectal teratoma is very rare and only 36 cases have been reported in the literature worldwide.[1] Majority of these cases are polypoid-shaped dermoid cysts protruding into the rectal lumen, like present case.

Dermoids are characteristically uniloculated cysts that have a lining comprising of special structures such as sebaceous glands, hair follicles, and teeth, and are filled with off-white, cheesy, sebaceous material.[4] But dermoids of the rectal wall can occur as either cysts or solid tumors.[2,5] Solid dermoid tumor without a cyst is a variant of mature teratoma or dermoid cyst that is even rarer type.[5]

A rectal dermoid may present with pain on defecation, per-rectal bleeding, and/or alteration in bowel habits. It may be per-rectally palpable as a mass arising from anterior or posterior wall of rectum or stalk can be felt with polypoidal growth protruding into lumen of rectum.[1] Some times, ovarian teratoma may rupture into rectum and presents with similar features.[6,7]

Transrectal ultrasound is used to assess rectal and perirectal masses because it is simple, accurate and inexpensive. CT scan may be useful for showing a cystic or solid mass in the perianal region and for helping in ruling out anal cancer or abscess. It may be seen on flexible sigmoidoscopy arising from anterior or posterior rectal wall and protruding into its lumen. Surface is usually smooth and glistening.[8]

Grossly, it may be cystic or solid with smooth glistening surface which is entirely covered with a fibrous and firm capsule, and is filled with an amorphous white creamy substance. Histological examination shows evidence of components from all three layers i.e. epidermal, mesodermal, and endodermal which is diagnostic of benign cystic teratoma. It may consist of a keratinizing stratified squamous epithelium with sebaceous glands and hair follicles.[1]

Malignant degeneration of a mature cystic teratoma is uncommon but has been reported. It is most commonly squamous cell carcinoma although adenosquamous has also been reported.[9] For the same reason, biopsy from the cystic lesions is not advisable as it may lead to spillage of malignant cells or infection of the cyst. Also complete excision of the cyst along with the margin of normal tissue followed by careful examination of the specimen is recommended.[10]

## CONCLUSION

Primary intrarectal dermoids are very rare. These are usually mature and cystic; solid ones are even rarer. It is usually benign but may turn malignant, so excisional biopsy is advisable. Present case was that of a solid intra-rectal dermoid arising from posterolateral rectal wall with a stalk that is exceedingly uncommon.

## References

[1] Sakurai Y, Uraguchi T, Imazu H, Hasegawa S, Matsubara T, Ochiai M (2000). Submucosal dermoid cyst of the rectum: Report of a case. Surg Today.

[2] Aldridge MC, Boylston AW, Sim AJ (1983). Dermoid cyst of the rectum. Dis Colon Rectum.

[3] Rao PL, Chary KS, Katariya RN (1979). Intrarectal dermoid: Report of a case. Aust N Z J Surg.

[4] Schueltz MJ, Elsheikh TM (2002). Dermoid cyst (mature cystic teratoma) of the cecum. Histologic and cytologic features with review of the literature. Arch Pathol Lab Med.

[5] Tabuchi Y, Tsunemi K, Matsuda T (1995). Variant type of teratoma appearing as a primary solid dermoid tumor in the rectum: Report of a case. Surg Today.

[6] Livesey SA, Conn PC, Dunn DC (1989). Benign cystic teratoma of the ovary rupturing into the rectum: A rare problem. Br J Clin Pract.

[7] Sasaki H, Nagasako K, Harada M, Kobayashi S, Uetake K (1979). Benign cystic teratoma of the ovary with rupture into the rectum: Report of a unique rectal tumor. Dis Colon Rectum.

[8] Green JB, Timmcke AE, Mitchell WT (1993). Endoscopic resection of primary rectal teratoma. Am Surg.

[9] Singh P, Yordan EL, Wilbanks GD, Miller AW, Wee A (1988). Malignancy associated with benign cystic terratomas (Dermoid cysts) of the ovary. Singapore Med J.

[10] Krivokapic, Dimitrijevic I, Barisic G, Markovic V, Krstic M (2005). Adenosquamous carcinoma arising within a retrorectal tailgut cyst: Report of a case. World J Gastroenterol.

